# Identification and Characterization of a New Bocavirus Species in Gorillas

**DOI:** 10.1371/journal.pone.0011948

**Published:** 2010-07-23

**Authors:** Amit Kapoor, Natasha Mehta, Frank Esper, Mateja Poljsak-Prijatelj, Phenix-Lan Quan, Natasha Qaisar, Eric Delwart, W. Ian Lipkin

**Affiliations:** 1 Center for Infection and Immunity, Columbia University, New York, New York, United States of America; 2 Rainbow Babies and Children's Hospital, Cleveland, Ohio, United States of America; 3 Faculty of Medicine, University of Ljubljana, Ljubljana, Slovenija; 4 Blood Systems Research Institute, University of California San Francisco, San Francisco, California, United States of America; Institut Pasteur, France

## Abstract

A novel parvovirus, provisionally named Gorilla Bocavirus species 1 (GBoV1), was identified in four stool samples from Western gorillas (*Gorilla gorilla*) with acute enteritis. The complete genomic sequence of the new parvovirus revealed three open reading frames (ORFs) with an organization similar to that of known bocaviruses. Phylogenetic analysis using complete capsid and non structural (NS) gene sequence suggested that the new parvovirus is most closely related to human bocaviruses (HBoV). However, the NS ORF is more similar in length to the NS ORF found in canine minute virus and bovine parvovirus than in HBoV. Comparative genetic analysis using GBoV and HBoV genomes enabled characterization of unique splice donor and acceptor sites that appear to be highly conserved among all four HBoV species, and provided evidence for expression of two different NS proteins in all primate bocaviruses. GBoV is the first non-human primate bocavirus identified and provides new insights into the genetic diversity and evolution of this highly prevalent and recently discovered group of parvoviruses.

## Introduction

Parvoviruses, members of family *Parvoviridae*, are small, non-enveloped icosahedral viruses with single-stranded linear DNA genomes that frequently infect animals through the fecal-oral route[1]. Parvoviruses are ubiquitous and can cause a broad spectrum of diseases in animals, including enteritis, panleukopenia, hepatitis, erythrocyte aplasia, immune complex–mediated vasculitis, and cerebellar ataxia [1]. Efficacious vaccines against animal parvovirus infections are widely employed [2], [3], [4], [5], [6].

The family *Parvoviridae* as currently defined, comprises two sub-families, *Densovirinae* and *Parvovirinae*, that infect non-vertebrate and vertebrate hosts, respectively[1]. The International Committee on Taxonomy of Viruses (ICTV) has further classified the sub-family Parvovirinae into five genera: Dependovirus, Bocavirus, Erythrovirus, Parvovirus and Amdovirus. Bocaviruses are unique among parvoviruses, as they contain a third ORF between non-structural and structural coding regions [7]. The genus Bocavirus includes the bovine parvoviruses, canine minute viruses and four species of human bocaviruses (HBoV1 -4)[1], [8], [9], .

HBoV1 infection has been linked with mild to severe lower respiratory tract infections in children, frequently in association with other viral infections [8], [13], [14], [15], [16], [17], [18], [19], [20]. HBoV1 has also been detected at low frequency in stool samples although association with diarrhea appears weaker than with respiratory symptoms [9], [10], [21], [22], [23], [24]. HBoV1 strains show a low degree of genetic variability world-wide (35,36). HBoV2 was recently identified in stool samples of Pakistani children [12]. Lower frequencies of HBoV2 were detected in the stool of Scottish adults and children [12]. A third bocavirus species, HBoV3, as well as HBoV2, were recently found in stool samples from Australian children with diarrhea [10]. HBoV4 was first identified in stool samples of children from Nigeria and Tunisia[11].

Group-reactive or pan-PCR approaches have been used to identify new viruses in human, animal and environmental samples [25], [26], [27], [28], [29]. We recently discovered two novel HBoV species using this approach [11]. In a survey for divergent bocaviruses in other non-human primates, we studied stool samples from three groups of western gorillas (*Gorilla gorilla*) with enteritis using pan-bocavirus PCR assay. This work describes the nearly complete genome of the first non-human primate bocavirus species identified in four stool samples of gorillas, tentatively named Gorilla Bocavirus species 1 (GBoV1).

## Materials and Methods

### Samples and source

All stool samples studied were from western gorillas (Gorilla gorilla) in a captive colony in North America. Samples were collected during April 2009, during an outbreak of enteritis resulting in diarrhea among three different groups of Gorillas. Group A = 12 animals, group B = 7 animals and group C = 4 animals. All fecal material was recovered from the floor of zoo enclosures. Samples were identified by group of origin, but the identity of the individual from which the sample was obtained was not always known.

### Sample processing and pan-bocavirus PCR assay

Stool samples were diluted 1∶9 with Hank's Buffered Saline Solution (Gibco-BRL), mixed with glass beads, vigorously vortexed and centrifuged twice at 2700 g for 10 min. Clarified supernatants (250 µl) were used for total nucleic acid extraction using the NucliSENS easyMAG system (Biomerieux). Total nucleic acids were eluted in 50 µl DEPC-treated water. PCR primers panBOV-F1 (5′-TAATGCAYCARGAYTGGGTIGANCC -3′) and panBOV-R1 (5′- GTACAGTCRTAYTCRTTRAARCACCA-3′) were used for the first round of hemi-nested PCR; while primers panBOV-F2 (5′- GCAYCARGAYTGGGTIGANCCWGC – 3′) and panBOV-R1 (same as first round) were used for the second round of hemi-nested PCR. For the first round of nested PCR, 5 µl of each specimen DNA was mixed with 5.2 µl 10× polymerase reaction buffer (Qiagen), 1.2 µl each dNTP (10 mM), 50 pmol forward (both panBOV-F1) and reverse primer (panBOV-R1), 1 µl HotStart Taq DNA polymerase (Qiagen) and 31 µl DEPC-treated water, in a total reaction volume of 50 µl.

The reaction was performed using initial denaturation at 95 C for 7 min, followed by six cycles of 95°C for 40 sec, 60°C for 45 sec and 68°C for 30 sec, then 35 cycles of 95°C for 30 sec, 57°C for 30 sec and 68°C for 30 sec, and final extension at 72°C for 10 min. During the first round of PCR, the first six cycles were done at a high annealing temperature to increase stringency and then remaining cycle at lower temperature to facilitate primer hybridization by tolerating some nucleotide mismatches. For the second round of hemi-nested PCR, identical cycling conditions were used, with an annealing temperature of 64°C for the first six cycles and annealing temperature of 58°C for the remaining 35 cycles. The reaction mixture for the second round was added with 0.5 µl PCR product from the first round. Products were visualized following electrophoresis on 2% agarose gel. PCR products showing positive bands of approximately 290 bp, corresponding to the highly conserved amplified NS gene fragment, were purified using a PCR purification kit (Qiagen) and directly sequenced from both directions.

To increase the sensitivity of the PCR specific primers targeting the same conserved gene region were used to screen all 23 samples. PCR primers GBoV-F1 (5′- GCACCAAGACTGGGTGGAACC – 3′) and GBoV-R1(5′- GCACCAGTGTAGTAGAGCTGC- 3′) were used for the first round of nested PCR. Primers GBoV-F2 (5′- CAAGACTGGGTGGAACCAGC-3′) and GBoV-R2(5′- GCACCAGTGTAGTAGAGCTGCAAT- 3′) were used for the second round. For the first round of nested PCR, 5 µl of each specimen DNA was mixed with 5.2 µl 10× polymerase reaction buffer (Qiagen), 1.2 µl each dNTP (10 mM), 20 pmol forward (GBoV-F1) and reverse primer (GBoV-R1), 1 µl HotStart Taq DNA polymerase (Qiagen) and 31 µl DEPC-treated water, in a total reaction volume of 50 µl. The reaction was performed using initial denaturation at 95 C for 7 min, followed by six cycles of 95°C for 40 sec, 61°C for 45 sec and 72°C for 30 sec, then 35 cycles of 95°C for 30 sec, 59°C for 30 sec and 72°C for 30 sec, and final extension at 72°C for 10 min. For the second round of nested PCR, identical cycling conditions were used, with an annealing temperature of 60°C for the first six cycles and annealing temperature of 58°C for the remaining 35 cycles. The reaction mixture for the second round was added with 0.5 µl PCR product from the first round. Products were visualized following electrophoresis on 2% agarose gel. PCR products showing positive bands of approximately 290 bp, corresponding to the highly conserved amplified NS gene fragment, were purified using a PCR purification kit (Qiagen) and directly sequenced from both directions.

### Complete genome sequencing and phylogenetic analysis

Complete genome of the GBoV1 was acquired using primer walking approach as previously described [11]. In brief, each extension step of PCR used one primer specific for the novel virus sequence and other degenerated to hybridize all known bocavirus sequences. After the complete genome was assembled, each base is sequenced in triplicate to confirm the sequence. The near complete genome of GBoV1 is submitted to Genbank under accession number HM145750.

To determine the sequence relationship between GBoV1 and other known bocavirus species, at least one representative virus member, including the reference genome from each species and their translated protein sequences, were used for generating sequence alignments. Sequences used for the comparison comprised the following: HBoV1 (GenBank accession no. 196168955, 161137732, 149389636, 125616893, 161137737, 149389621, 149389676, 223670836, 196168950, 149389626, 284799140, 256692975, 149389721, 149389656, 241995009, 223951374, 196169010, 149389641, 92087153, 226434473, 223670876, 223670861, 196169000, 196168975, 196168960, 149389651, 149389611, 66356133, 66356128, 219879196, 196168985, 196168970, 196168945, 149389711, 149389706, 149389696, 149389661, 149389646, 149389606, 125616883, 196168995, 149389701, 149389691, 149389681, 149389671, 149389666, 149389631, 130918044, 84873482, 260779772, 256692980, 226434468, 223670851, 223670846, 223670841, 223670831, 125719360, 226434443), HBoV2 (GenBank accession no. 241994927, 213012741, 237690160, 237690166, 158714105, 238476986, 158713998, 290565732, 213012746, 290565722, 213012751, 284080644, 237690154, 284080639, 290565727), HBoV3 (GenBank accession no. 238476991,206572554,290565737,237690178,237690184), HBoV4 (GenBank accession no. 289163341), canine minute virus (GenBank accession no. NC_004442) and bovine parvovirus-1 (GenBank accession no. 9627190). Phylogenetic analyses of CLUSTAL W aligned regions were carried out by neighbour joining of nucleotide or amino acid p-distances, implemented in the program MEGA4[30]. Bootstrap resampling was carried out to demonstrate robustness of groupings. Recombination analysis was performed by Symplot[31], using reference genomes of all four HBoV species and GBoV1. To assess similarity across the genomes, sequence scans were performed using a fragment length of 300 bases and an increment of bases between fragments.

## Results

### Identification and near complete genome of GBoV1

Twenty-three stool samples collected during an outbreak of enteritis with diarrhea in captive gorillas were studied for presence of divergent bocaviruses related to known HBoV. Four samples (two each from group B and C) yielded amplification products using a pan-bocavirus PCR. To increase the sensitivity of the PCR specific primers targeting GBoV1 sequence were used to screen all samples, but no additional samples were found positive. Preliminary sequence analysis confirmed presence of a divergent bocavirus with 89% nucleotide and 92% amino acid identity to HBoV1. The causal relationship between the virus and disease was not determined.

Similar to other known bocaviruses, the GBoV1 genome is predicted to contain three large ORFs. ORF1 encodes nonstructural (NS) protein; ORF2 codes for overlapping VP1/VP2 capsid proteins; ORF3 encodes NP1. The GBoV1NS protein gene comprises 2412 nt and is predicted to code for 804 amino acids (aa) with a mass of approximately 91 kDa, making it the second longest NS protein encoded by a bocavirus (HBoV1 – 781 aa, HBoV2 -777 aa, HBoV3 – 780 aa and HBoV4 – 776 aa, canine minute virus - 774 aa and bovine parvovirus - 860 aa). Conserved motifs associated with rolling circle replication, helicase and ATPase are present within ORF1. The GBoV1 virion protein gene comprises 2013 nt and is predicted to code for two structural proteins VP1 and VP2 of 671 aa and 542aa length, respectively. Within ORF2, the phospholipase A2 motifs required for parvovirus infectivity situated within the VP1-unique (VP1u) region were also found in association with calcium-binding loop and catalytic residues. The occurrence of the non-structural protein NP1 encoded by an ORF in the middle of the genome is a unique feature of bocaviruses. The GBoV NP1 gene comprises 657 nt and is predicted to code for a highly basic and stable 219 aa protein with a mass of approximately 26 kDa. NP1 is a highly phosphorylated protein of currently undetermined function [32]; NP1 differs in length between different HBoV species, ranging from 219 aa (HBoV1), 214 aa (HBoV2A1, HBoV3), 215 aa (HBoV1A2, HBoVA3, HBoVB) and 218 aa (HBoV4).

### Phylogenetic relationship of GBoV1 with other bocaviruses

**Figure 1:**
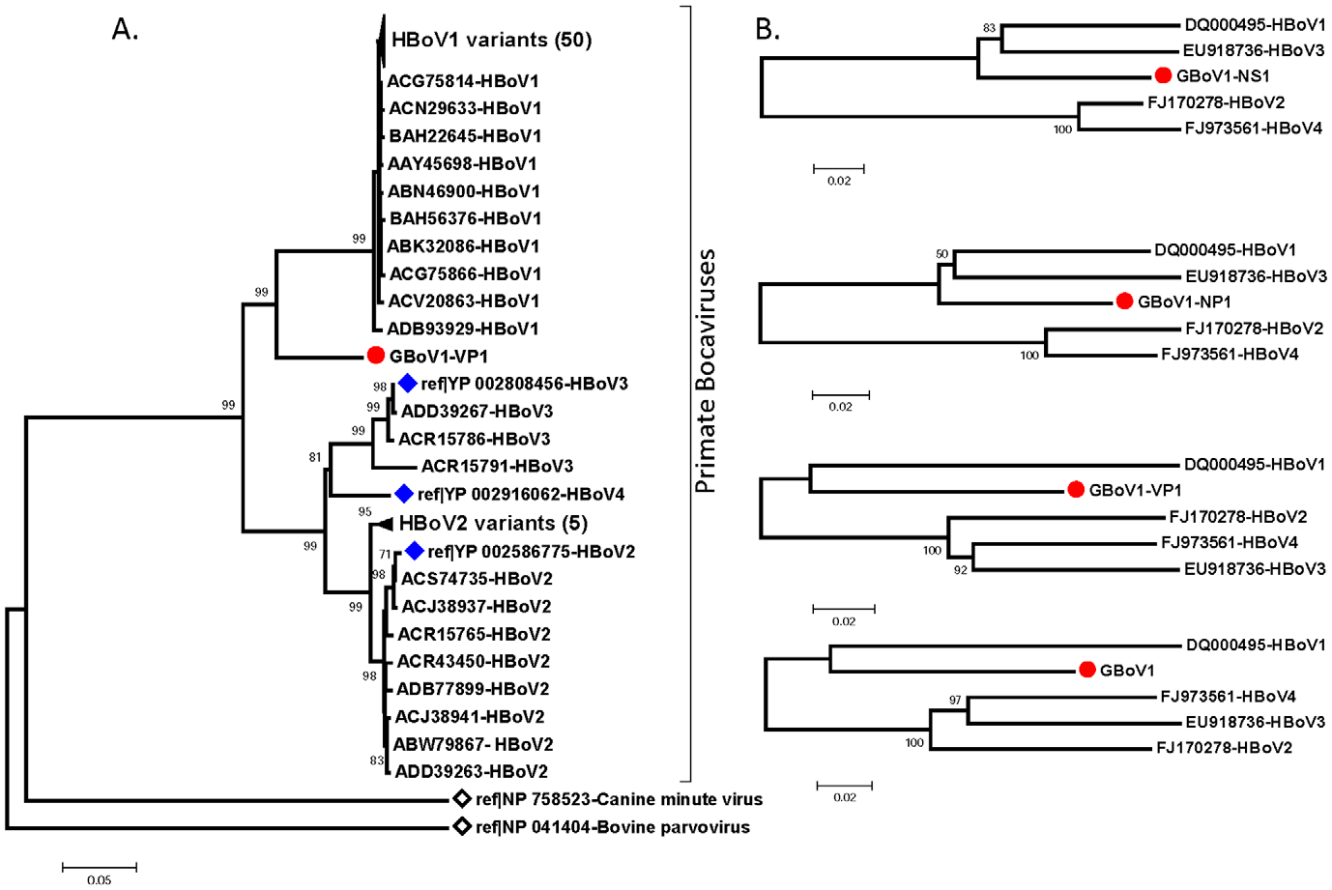
Phylogenetic analysis of GBoV1. (A) Full length structural protein (VP1/2) sequences of all HBoV and animal bocavirus strains available in GenBank were used to determine phylogeny of GBoV1 by neighbor-joining analysis of pairwise distances between translated amino acid sequences. Bootstrap re-sampling was used to determine robustness of individual clades (values above 70% shown above the branches). (B) The 4 major open reading frames of GBoV1 were analyzed using maximum likelihood composition analysis method (MEGA4.1) comparing pairwise distances of translated sequences of representative variants (reference sequence) of all four HBoV species. Accession numbers of sequences used precedes the name of corresponding bocavirus species.

To determine the relationship between GBoV1 and other members of the *Bocavirus* genus, phylogenetic analyses of the 3 large ORFs - NS1, NP1 and VP1/VP2 - were performed, by use of both nt and deduced aa sequences. In all 3 genomic regions, the GBoV1 was more similar to HBoV1 or HBoV3 than to the other human or animal bocavirus species (CnMV and BPV1), the latter of which consistently occupied an outlier position with regard to the clade containing all primate bocavirus sequence (Fig.1 A and B).

Most HBoV species include several genotypes. Thus, to ensure accurate classification of GBoV1 we examined complete capsid sequences of all the HBoV variants in the GenBank. Phylogenetic analyses suggested that although GBoV1 is genetically closest to HBoV1, the distance between the two viruses is more than that observed within (intra-species distance) any of the four reported HBoV species (Fig.1 A)[11]. Moreover, as HBoV1 was the most genetically homogenous bocavirus species, GBoV1 is more likely to represent a separate genetic clade. Phylogenetic clustering of primate bocaviruses using the different genes was found to be consistent except for HBoV3, which being a recombinant virus[11], clustered in NS gene with HBoV1 clade and in other genes with HBoV2 clade (Fig.1-B). GBoV1 was genetically more similar to HBoV1 in all genes, suggesting it is not an inter-species recombinant virus (Fig.1-B). Phylogenetic trees using different genes also showed that the genetic distance of GBoV1 and its closest related virus (HBoV1) was more than that between- HBoV2 and 4 (in all genes), as well between HBoV2, 3 and 4 (in VP1 and VP2 genes) (Fig.1-B). These relationships are described in Table 1 in terms of pairwise nt and aa distances. Differences between GBoV1 and HBoV1 and HBoV2 in VP1/2 genes were substantially greater (12.1–12.8 and 13.1–14%, respectively) than those between either HBoV2 and HBoV3 (9.2–10.5 and 9.4–10.8%) or HBo2 and HBoV4 (9.6–11.4 and 9.6–11.3%) or HBoV3 and HBoV4 (7.8–8.8 and 7.9–8.9%). However the genetic distance between the VP1/2 gene of HBoV1 and HBoV2 (19.6–21.8 and 20.3–20.5) was the largest observed between primate bocavirus species (Table 1, shaded numbers).

**Table 1:** Comparison of pairwise nucleotide and amino acid (bold typeface) distances (p-distance) of all four genes between GBoV1 and HBoV species confirms GBoV1 as prototype of a new bocavirus species.

	GBoV1	HBoV1	HBoV2	HBoV3	HBoV4
**GBoV1**					
NSNPVP1VP2	----	7.812.812.112.8	24.230.717.218.9	8.916.116.918.9	25.028.316.718.7
HBoV1					
NSNPVP1VP2	**8.0** **12.8** **13.1** **14.0**	----	21.531.619.621.8	8.516.619.421.7	22.330.219.121.7
HBoV2					
NSNPVP1VP2	**24.3** **31.3** **18.0** **19.6**	**21.8** **31.8** **20.3** **22.5**	----	23.529.99.210.5	5.711.39.611.4
HBoV3					
NSNPVP1VP2	**8.9** **16.1** **17.4** **19.1**	**8.6** **16.5** **19.8** **21.9**	**23.6** **30.8** **9.4** **10.8**	----	24.530.87.88.8
HBoV4					
NSNPVP1VP2	**25** **29.0** **17.2** **19.0**	**22.5** **30.8** **19.5** **21.9**	**5.6** **12.1** **9.6** **11.3**	**24.6** **31.8** **7.9** **8.9**	----

The International Committee on Taxonomy of Viruses (ICTV) criteria for classification of Bocaviruses establishes that members of each species are probably antigenically distinct and that natural infection is confined to a single host species. Species are defined as <95% homologous NS gene DNA sequence (http://www.ncbi.nlm.nih.gov/ICTVdb/ICTVdB/or http://phene.cpmc.columbia.edu/Ictv/fs_parvo.htm). While the antigenic properties of GBoV1 were not studied here, its isolation from a different natural host (gorilla) and >5% genetic distance in NS gene compared to other known bocavirus species, suggest that GBoV1 should be classified as prototype virus of a new bocavirus species.

### Unique genomic features of GBoV1 and recombination analysis

**Figure 2:**
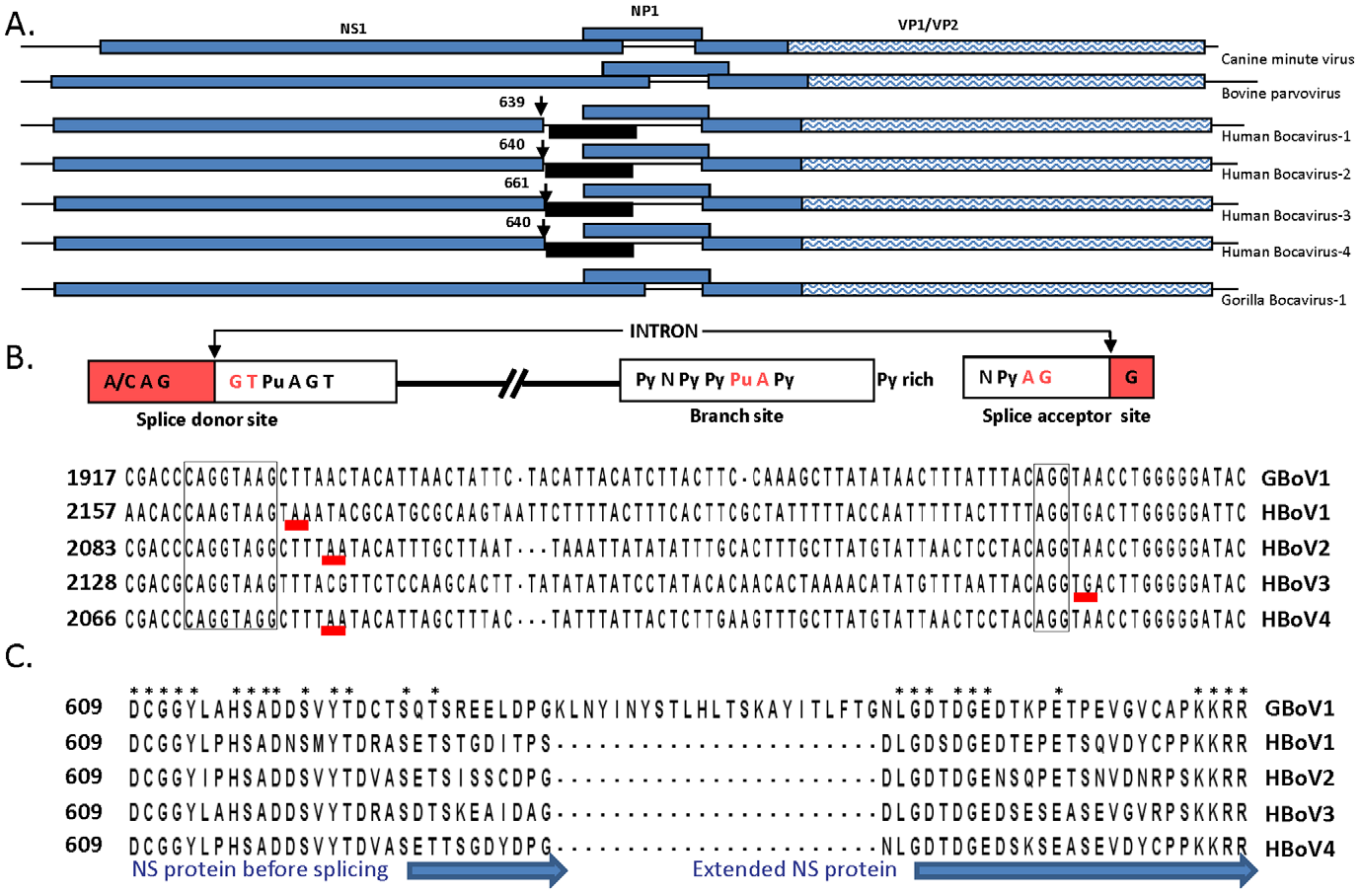
Genomic organization of primate bocaviruses. (A) Shows genomic organization of known primate and animal bocavirus species. Small arrows indicate positions (numbers) of stop codons in NS gene of human bocaviruses. (B) Shows consensus eukaryotic RNA splicing sequence and presence of similar sequence motifs found conserved in NS genes of all primate bocaviruses. Numbers represent nucleotide positions on respective genomes; stop codons found in HBoV species are underlined in red. No stop codon was found in the NS1 gene region of GBoV1. (C) Shows presence of substantive protein identity between the extended NS protein of HBoVs and longer uninterrupted NS protein of GBoV1. Numbers represent relative amino acid positions of different bocavirus NS proteins.

The genomic organization of all four HBoV species is remarkably similar to that of animal bocaviruses, except that their ORF1 encodes a shorter NS protein (639–650 aa), compared to the longer NS protein of animal bocaviruses (770–860 aa) (Fig.2A). We recently showed the presence of highly conserved potential RNA splicing signals in all HBoV species near the end of the smaller NS2 ORF and the putative second exon encoding the C-terminal region of NS1 (Fig. 2B)[11]. We reported that the putative elongated NS1 resulting from such a spliced transcript in HBoV encodes a 775–781 aa protein with a carboxy terminus that has weak similarity (25–34% aa identity) to that of other, non-human bocaviruses. Interestingly, the putative NS protein of GBoV1, which is predicted to arise from an intact (non-spliced) transcript, has higher protein similarity (58–74% aa identity) to the extended NS protein (post splicing transcript) of all HBoVs (Fig.2C). Moreover, although no stop codon was found in NS gene of GBoV1, the elements required for RNA splicing were present GBoV1 as in all HBoV species (Fig. 2C, splice donor, branch and acceptor site). Analysis of recently reported novel HBoV species (HBoV3) showed it as a recombinant virus of HBoV1 and HBoV2, suggesting interspecies recombination among bocaviruses. We compared genome wide distances among HBoV1, HBoV2 and GBoV1 genomes using Symplot analysis [31] but found no evidence of interspecies recombination. However, comparison including HBoV3 and HBoV4 genomes indicates frequent recombination between all primate species (data not shown).

## Discussion

We report the identification and near complete genomic sequencing of the first non-human primate bocavirus. As it was found in stool samples of gorillas, we have provisionally named this virus GBoV1. Following ICTV guidelines we propose classification of this novel virus as the prototype member of a new species in the genus Bocavirus [1]. The pan-PCR approach used to discover GBoV1 was similar to that recently used to identify HBoV3 and HBoV4 [11].

Several studies report higher prevalence of HBoV1 in respiratory sample and of HBoV2, 3 and 4 in gastrointestinal samples, suggesting that different species may vary in tissue tropism[7], [9], [10], [11], [12], [13], [14], [16], [19], [21], [22], [33], [34], [35], [36], [37], [38], [39], [40], [41]. We do not know whether GBoV1 shares this propensity to tissue tropism. Comparative genetic analysis of different HBoV strains circulating worldwide indicate that whereas HBoV1 is homogenous with low mean genetic diversity (<1% nt or <0.5% aa), HBoV2 is heterogeneous with higher mean genetic diversity (<4.6% nt or <6.1% aa)[11]. Current data do not allow us to determine the genetic diversity of GBoV1. However, the discovery of GBoV1 indicates that this recently discovered group of viruses are more widely dispersed than previously reported and raises the possibility that more novel viral species may be found in other animal species.

A unique feature of the GBoV1 genome was the presence of a longer NS protein ORF compared to that encoded by most closely related HBoV. Significant and high similarity (58 to 74% aa identity) of the extended NS protein of all HBoV species with GBoV1 NS protein supports expression of two NS proteins of variable length in HBoVs [11]. The proposed NS1 RNA splicing and NS1 protein expression itself were detected using Northern blots and NS1 C-termini specific sera, respectively, in 293 and human epithelial cells transfected with plasmids expressing HBoV1 transcripts [42]. Despite their high genetic diversity, similar to all HBoV species, the GBoV1 genome also has perfectly conserved NS gene RNA splicing sites, suggestive of expression of two different NS proteins in GBoV. However it is unclear whether only HBoV requires expression of a truncated NS protein and whether the presence of a truncated NS protein is advantageous for HBoV replication in humans.

Parvoviruses have the capacity to change host species [2], [43]. The observation that GBoV1 is more closely related to HBoV1 than any other two HBoV species are to one another, suggests the possibility of cross-species transmission. Single-stranded DNA parvoviruses have been shown to have a mutation rate approaching that of RNA viruses [44], [45]. Moreover, both the expression of a truncated NS protein (presence of stop cordon in all HBoV species) and the presence of splicing mechanism that generates a longer NS protein is unique to all HBoV species, and suggests the derivation of all HBoV species from a common ancestor. We conclude that although interspecies transmission of bocaviruses may have occurred in ancient times, the genomic organization of GBoV1 indicates that there has been species specificity of bocaviruses in primates for a long time. In absence of any known animal model for HBoV, identification of GBoV1 and other non-human primate bocaviruses will offer opportunity to study the HBoV pathogenesis and disease.

## References

[1] Claude M, Fauquet MAM, Maniloff J, Desselberger U, Ball LA (2004). Virus Taxonomy: The Eighth Report of the International Committee on Taxonomy of Viruses..

[2] Hoelzer K, Parrish CR The emergence of parvoviruses of carnivores.. Vet Res.

[3] Schultz RD (2006). Duration of immunity for canine and feline vaccines: a review.. Vet Microbiol.

[4] Paul MA, Carmichael LE, Childers H, Cotter S, Davidson A (2006). 2006 AAHA canine vaccine guidelines.. J Am Anim Hosp Assoc.

[5] Patel JR, Heldens JG (2009). Review of companion animal viral diseases and immunoprophylaxis.. Vaccine.

[6] Appel MJ (1999). Forty years of canine vaccination.. Adv Vet Med.

[7] Manteufel J, Truyen U (2008). Animal bocaviruses: a brief review.. Intervirology.

[8] Allander T, Tammi MT, Eriksson M, Bjerkner A, Tiveljung-Lindell A (2005). Cloning of a human parvovirus by molecular screening of respiratory tract samples.. Proc Natl Acad Sci U S A.

[9] Anderson LJ (2007). Human bocavirus: a new viral pathogen.. Clin Infect Dis.

[10] Arthur JL, Higgins GD, Davidson GP, Givney RC, Ratcliff RM (2009). A novel bocavirus associated with acute gastroenteritis in Australian children.. PLoS Pathog.

[11] Kapoor A, Simmonds P, Slikas E, Li L, Bodhidatta L Human bocaviruses are highly diverse, dispersed, recombination prone, and prevalent in enteric infections.. J Infect Dis.

[12] Kapoor A, Slikas E, Simmonds P, Chieochansin T, Naeem A (2009). A newly identified bocavirus species in human stool.. J Infect Dis.

[13] Allander T (2008). Human bocavirus.. J Clin Virol.

[14] Allander T, Jartti T, Gupta S, Niesters HG, Lehtinen P (2007). Human bocavirus and acute wheezing in children.. Clin Infect Dis.

[15] Bastien N, Chui N, Robinson JL, Lee BE, Dust K (2007). Detection of human bocavirus in Canadian children in a 1-year study.. J Clin Microbiol.

[16] Catalano-Pons C, Bue M, Laude H, Cattan F, Moulin F (2007). Human bocavirus infection in hospitalized children during winter.. Pediatr Infect Dis J.

[17] Chung JY, Han TH, Kim CK, Kim SW (2006). Bocavirus infection in hospitalized children, South Korea.. Emerg Infect Dis.

[18] Fry AM, Lu X, Chittaganpitch M, Peret T, Fischer J (2007). Human bocavirus: a novel parvovirus epidemiologically associated with pneumonia requiring hospitalization in Thailand.. J Infect Dis.

[19] Gagliardi TB, Iwamoto MA, Paula FE, Proenca-Modena JL, Saranzo AM (2009). Human bocavirus respiratory infections in children.. Epidemiol Infect.

[20] Lau SK, Yip CC, Que TL, Lee RA, Au-Yeung RK (2007). Clinical and molecular epidemiology of human bocavirus in respiratory and fecal samples from children in Hong Kong.. J Infect Dis.

[21] Albuquerque MC, Rocha LN, Benati FJ, Soares CC, Maranhao AG (2007). Human bocavirus infection in children with gastroenteritis, Brazil.. Emerg Infect Dis.

[22] Campe H, Hartberger C, Sing A (2008). Role of Human Bocavirus infections in outbreaks of gastroenteritis.. J Clin Virol.

[23] Lindner J, Modrow S (2008). Human bocavirus–a novel parvovirus to infect humans.. Intervirology.

[24] Ziegler S, Tillmann RL, Muller A, Simon A, Schildgen V (2008). No gastroenteric Bocavirus in high risk patients stool samples.. J Clin Virol.

[25] Oberste MS, Maher K, Nix WA, Michele SM, Uddin M (2007). Molecular identification of 13 new enterovirus types, EV79-88, EV97, and EV100-101, members of the species Human Enterovirus B.. Virus Res.

[26] Oberste MS, Maher K, Michele SM, Belliot G, Uddin M (2005). Enteroviruses 76, 89, 90 and 91 represent a novel group within the species Human enterovirus A.. J Gen Virol.

[27] Wellehan JF, Childress AL, Marschang RE, Johnson AJ, Lamirande EW (2009). Consensus nested PCR amplification and sequencing of diverse reptilian, avian, and mammalian orthoreoviruses.. Vet Microbiol.

[28] Kapoor A, Victoria J, Simmonds P, Slikas E, Chieochansin T (2008). A highly prevalent and genetically diversified Picornaviridae genus in South Asian children.. Proc Natl Acad Sci U S A.

[29] Kapoor A, Li L, Victoria J, Oderinde B, Mason C (2009). Multiple novel astrovirus species in human stool.. J Gen Virol.

[30] Kumar S, Nei M, Dudley J, Tamura K (2008). MEGA: a biologist-centric software for evolutionary analysis of DNA and protein sequences.. Brief Bioinform.

[31] Lole KS, Bollinger RC, Paranjape RS, Gadkari D, Kulkarni SS (1999). Full-length human immunodeficiency virus type 1 genomes from subtype C-infected seroconverters in India, with evidence of intersubtype recombination.. J Virol.

[32] Lederman M, Patton JT, Stout ER, Bates RC (1984). Virally coded noncapsid protein associated with bovine parvovirus infection.. J Virol.

[33] Brieu N, Guyon G, Rodiere M, Segondy M, Foulongne V (2008). Human bocavirus infection in children with respiratory tract disease.. Pediatr Infect Dis J.

[34] Chieochansin T, Samransamruajkit R, Chutinimitkul S, Payungporn S, Hiranras T (2008). Human bocavirus (HBoV) in Thailand: clinical manifestations in a hospitalized pediatric patient and molecular virus characterization.. J Infect.

[35] Chieochansin T, Thongmee C, Vimolket L, Theamboonlers A, Poovorawan Y (2008). Human bocavirus infection in children with acute gastroenteritis and healthy controls.. Jpn J Infect Dis.

[36] de Vries JJ, Bredius RG, van Rheenen PF, Smiers FJ, Scholvinck EH (2009). Human bocavirus in an immunocompromised child presenting with severe diarrhea.. J Clin Microbiol.

[37] Gerna G, Piralla A, Campanini G, Marchi A, Stronati M (2007). The human bocavirus role in acute respiratory tract infections of pediatric patients as defined by viral load quantification.. New Microbiol.

[38] Kahn J (2008). Human bocavirus: clinical significance and implications.. Curr Opin Pediatr.

[39] Schildgen O, Muller A, Allander T, Mackay IM, Volz S (2008). Human bocavirus: passenger or pathogen in acute respiratory tract infections?. Clin Microbiol Rev.

[40] Schildgen O, Muller A, Simon A (2007). Human bocavirus and gastroenteritis.. Emerg Infect Dis.

[41] Vicente D, Cilla G, Montes M, Perez-Yarza EG, Perez-Trallero E (2007). Human bocavirus, a respiratory and enteric virus.. Emerg Infect Dis.

[42] Chen AY, Cheng F, Lou S, Luo Y, Liu Z Characterization of the gene expression profile of human bocavirus.. Virology.

[43] Parrish CR, Kawaoka Y (2005). The origins of new pandemic viruses: the acquisition of new host ranges by canine parvovirus and influenza A viruses.. Annu Rev Microbiol.

[44] Shackelton LA, Parrish CR, Truyen U, Holmes EC (2005). High rate of viral evolution associated with the emergence of carnivore parvovirus.. Proc Natl Acad Sci U S A.

[45] Hoelzer K, Shackelton LA, Parrish CR, Holmes EC (2008). Phylogenetic analysis reveals the emergence, evolution and dispersal of carnivore parvoviruses.. J Gen Virol.

